# Can we spice up our Christmas dinner? Busting the myth of the ‘Chinese restaurant syndrome’

**DOI:** 10.1007/s12471-017-1053-5

**Published:** 2017-11-10

**Authors:** N. W. E. van den Berg, J. Neefs, W. R. Berger, S. W. E. Baalman, E. Meulendijks, M. Kawasaki, E. M. Kemper, F. R. Piersma, M. W. Veldkamp, R. Wesselink, S. P. J. Krul, J. R. de Groot

**Affiliations:** 1 Heart Centre, Departments of Cardiothoracic Surgery and Cardiology, Academic Medical Centre, University of Amsterdam, Amsterdam, The Netherlands; 2 Department of Hospital Pharmacy, Academic Medical Centre, University of Amsterdam, Amsterdam, The Netherlands

**Keywords:** Chinese restaurant syndrome, Atrial fibrillation, MSG

## Abstract

**Background:**

Monosodium glutamate (MSG), also referred to as Vetsin or E621, is a flavour enhancer frequently used in Asian cuisine and abundantly present in the famous Chinese dish *Peking duck.* MSG is notorious for triggering the onset of the so-called ‘Chinese restaurant syndrome’ (CRS), a complex of unpleasant symptoms, which might include flushing, sweating and the onset of atrial fibrillation (AF). This study aims to determine the effects of MSG on the occurrence of AF.

**Methods:**

We conducted a placebo self-controlled single-arm study in the Academic Medical Centre in Amsterdam. We included paroxysmal AF patients who reported a consistent onset of AF upon MSG intake. During three admissions, participants were subsequently administered: placebo, 1.5 g and 3 g MSG. If AF was recorded after the dose of 1.5 g MSG, patients were given another placebo instead of 3 g MSG. The primary outcome was the onset of AF registered by 24-hour Holter monitoring. The secondary outcomes were any other arrhythmia and the onset of CRS defined as two or more symptoms of CRS after MSG intake.

**Results:**

Six men participated in the study. Both 1.5 g and 3 g MSG were unrelated to CRS, arrhythmias or AF occurrence.

**Conclusion:**

*Peking duck* can be put on the Christmas menu without risking guests to be admitted to the emergency department with new episodes of AF.

**Electronic supplementary material:**

The online version of this article (10.1007/s12471-017-1053-5) contains study inlcusion and exclusion criteria and recipe of Peking duck, which is available to authorized users.

## What this study adds

Many Dutch people are aware of, or have experience with, the fact that spicy food may cause a wide range of symptoms such as sweating or palpitations. This so-called ‘Chinese restaurant syndrome’ is thought to be caused by monosodium glutamate (MSG) and may trigger AF onset. Our study is the first placebo-controlled trial to study the effects of different doses of MSG. From our data, we cannot proof that MSG is associated with AF in self-reported MSG-sensitive patients.

## Introduction

The Chinese restaurant syndrome is known for its unpleasant symptoms following the consumption of spicy food, including headache, sweating, palpitations and the onset of atrial fibrillation (AF), which is the most common cardiac arrhythmia. The Chinese restaurant syndrome was first described in 1968 by a doctor who reported symptoms directly after the consumption of Chinese food, but without a ‘hangover effect’ [1]. Later, the physical reactions of the Chinese restaurant syndrome were attributed to the flavour enhancer monosodium glutamate (MSG), commonly known in Asian cuisine as E621, and popularly known as Vetsin [2, 3]. MSG is present in countless food products and flavour enhancers and is likely to be on many Christmas menus inadvertently, exposing many families to the risk of a truly unforgettable Christmas Eve. This thus warrants further investigation.

Several tabloids and magazines advise patients with a history of AF to avoid spicy food containing MSG. A Google search for ‘MSG products’ returns quotes such as ‘*danger to your health’*, ‘*slow poison*!’ or ‘*a silent killer’*. Physicians treating AF patients are likely to have encountered the undisputed self-reported linkage by patients between specific foods and AF onset. Not infrequently, physicians advise patients to stay off MSG-containing foods, although most of the proof that links MSG to AF onset is described in tabloids and scientific evidence is limited to case reports and surveys [4, 5].

Meanwhile, experimental cell and mice studies assessed the pathophysiological pathways of MSG causing AF. AF onset was attributed to stimulation of the cardiac glutamate receptor by glutamate [6, 7], an important component of MSG that is believed to induce an increased heart rate and, potentially, palpitations [3, 8].

On the one hand, an irrevocable association between MSG and AF onset has not yet been demonstrated. Confirmation of the association could be used to put together a tasteful Christmas menu without the onset of the Chinese restaurant syndrome. More importantly, the association could be used by physicians for the counselling of AF patients throughout the rest of the year. On the other hand, the absence of an association could quell online gossip. Finally, it could revolutionary cast away the roast turkey with cranberry sauce and Christmas pudding from the Christmas menu, replacing it with *Peking duck*.

With this placebo-controlled trial we aimed to investigate the effect of different doses of MSG on the onset of arrhythmias in self-reported MSG-receptive patients.

## Methods

We conducted a placebo self-controlled single-arm study, including patients with a history of paroxysmal AF who reported a consistent onset of AF upon MSG intake (a complete list of study inclusion and exclusion criteria is provided in Table 1 of the online supplementary material). The study was approved by the Medical Ethics Committee of the Academic Medical Centre (registration ID: NL31448.018.10). All participants provided written informed consent.

**Fig. 1:**
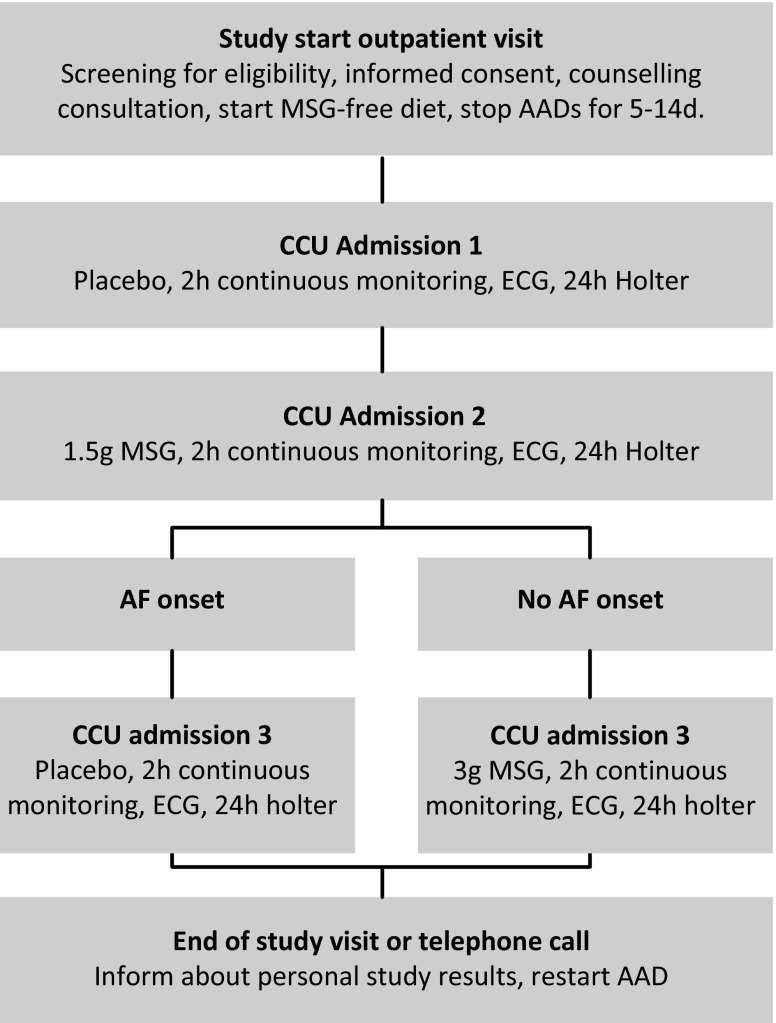
Flow chart of study design. *AADs* antiarrhythmic drugs, *AF* atrial fibrillation *CCU* coronary care unit, *ECG* electrocardiogram, *MSG* monosodium glutamate

Participants were admitted to the cardiac care unit for two hours rhythm monitoring on three occasions with one-week intervals. Participants were kept off anti-arrhythmic drugs and were self-evidently advised to stay away from the Chinese restaurant or from MSG-containing food products. During three admissions, participants were subsequently administered placebo, 1.5 g and 3 g MSG. If AF was recorded after the dose of 1.5 g MSG, patients were given another placebo instead of 3 g MSG (see study flowchart in Fig. 1). Study medication was prepared in a manner that enabled blinding of treatment allocation, as MSG has a very peculiar and tasty character called ‘umami’. Therefore, participants could serve as their own control. Participants were hospitalised for two hours with continuous rhythm monitoring to span the most critical 20–30 min typical for AF onset after MSG intake. Thereafter, they were discharged with 24-hour Holter monitoring.

The primary outcome was AF onset within 24 h after MSG intake. Secondary outcomes included any arrhythmia or symptom of the Chinese restaurant syndrome. The Chinese restaurant syndrome was considered present in case of two or more complaints. When participants suffered from symptoms both after placebo and after MSG, we considered it a negative result. If AF occurred, participants were treated according to the discretion of the treating provider. Baseline information was obtained from electronic medical patient records and a dietary questionnaire. For comparisons of continuous data, we used the unpaired sample T‑tests or the Mann-Whitney U test, when appropriate. Categorical data were compared by Fisher’s exact test. Two-sided *p*-value of <0.05 was considered to be significant. Data was analysed by means of IBM SPSS Statistics for Windows, version 24 (IBM Corp., Armonk, N. Y., USA) and R Version 3.3.2.

## Results

During an inclusion period of four years, eight patients were deemed eligible out of approximately 2,000 patients with AF visiting the outpatient clinic. We were able to include six patients with a history of paroxysmal AF and a self-reported onset of AF after MSG consumption, thereby yielding an impressively low inclusion rate of 1.5 inclusions per year.

Baseline characteristics of participants are presented in Table 1. Participants were all Caucasian men with a mean age of 58.5 (interquartile range (IQR): 55.8–65.0) years, who had been diagnosed with paroxysmal AF for a median duration of 6.5 (IQR: 2.3–10.0) years. All participants reported symptoms of AF after the consumption of accountable products. Furthermore, 100% reported palpitations. We were able to assess dietary habits in five patients. One patient did not fill out the questionnaire, because he emigrated to China. The five participants were on a Western diet. Food most often held accountable for AF onset was Chinese food (*n*: 5, 100%). Dried and processed tomatoes (*n*: 3, 60%) came in second place.

**Table 1:** Baseline characteristics of participants; (the total number of participants questioned varied between 5 and 6, due to one patient’s emigration to China)

	*N* (%)
Age, years median [IQR]	58.5 [55.8–65.0]
Gender, male (%)	6 (100)
Atrial fibrillation duration, years [IQR]	6.5 [2.3–10.0]
Alcoholic beverages consumed per week, (units)	5–10
*Reported complaints, n (%)*	
Atrial fibrillation	6 (100)
Dyspnoea	3 (60)
Sweating	5 (100)
Arousal	3 (60)
Hangover, *n* (%)	1 (16.7)
Delay until onset of symptoms, hours range	1–5
Personal measures taken	Avoiding MSG-containing foods, keeping an allergy list, informing hosts and restaurants beforehand, providing information on websites, carrying a magnifying glass to inspect product ingredient lists, examining ECGs with an oscillometer
Described dishes	Shrimps, mussels, tomatoes (especially when dried or processed), peas, beer, wine, spirits, chocolate, Dutch mature cheeses, instant soup, meat products, Döner kebab, instant noodles, pizza, spice mixes, crisps, ketchup, chili powder, paprika powder, Mexican food, Chinese food, Japanese food, Italian food, Middle Eastern food

*ECG* electrocardiogram, *IQR* interquartile range, *MSG* monosodium glutamate

In total, four episodes of AF were registered in two participants. In both participants, an episode of AF occurred after the intake of 1.5 g MSG, but also after the intake of placebo (Table 2). Outcome was thus considered negative. In the first participant with AF, both arrhythmia episodes started 6 h after either placebo or 1.5 g MSG intake. In the second participant, the arrhythmia episodes started 24 h after placebo intake and 14 h after 1.5 g MSG intake. The other patients did not demonstrate any arrhythmia upon repeated trials with 1.5 g and 3 g MSG.

**Table 2:** Study outcome per patient

	Visit 1	Visit 2	Visit 3
Participant 1	**Placebo**	**1.5 g MSG**	Lost
Participant 2	Placebo	1.5 g MSG	3 g MSG
Participant 3	Placebo	**1.5 g MSG** ^a^	**Placebo**
Participant 4	Placebo	1.5 g MSG	3 g MSG^b^
Participant 5	Placebo	1.5 g MSG	3 g MSG
Participant 6	Placebo	1.5 g MSG	Lost

*Bold* indicates AF onset; *AF* atrial fibrillation, *MSG* monosodium glutamate

^a^This occurrence of AF was reported after night-time activity

^b^This participant went to the Chinese restaurant after the intake of 3 g of MSG

**Fig. 2:**
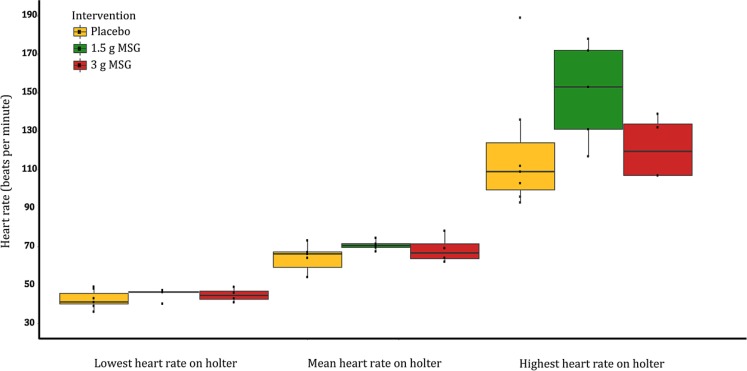
Heart rates on Holter monitoring for each intervention group. Mean heart rate, *p*-value: 0.59; low heart rate, *p*-value: 0.43; high heart rate, *p*-value: 0.31

Holter monitoring revealed a median heart rate of 67 (IQR: 64–70) beats per minute (bpm), with a minimum heart rate of 45 (IQR: 41–46) bpm and a maximum heart rate of 124 (IQR: 107–143) bpm. No difference in minimum, maximum or median heart rate was found after MSG intake compared to placebo (Fig. 2). No other arrhythmias or conduction disorders were detected. Heart rates were similar between the subjects with and without AF.

Two participants who reported palpitations during follow-up suffered from AF at the time of the complaints. No other complaints were reported, therefore, no participant reached the criteria for the Chinese restaurant syndrome.

## Discussion

Despite persistent warnings for the Chinese restaurant syndrome, we found no difference in AF occurrence after the intake of 1.5 g or 3 g MSG compared with placebo. The episodes of AF were registered after both the intake of MSG and placebo. Moreover, AF did not occur during the typical time window of the syndrome [9]. There was no difference in self-reported symptoms or heart rate during follow-up. The study population consisted of only males, possibly explained by the higher prevalence of AF amongst men. Only eight patients reported an association between MSG and the onset of AF, and were thus deemed eligible. Six participants were included out of 2,000 AF patients during a four-year inclusion period, tentatively suggesting an imbalance between the attributions to the syndrome and the occurrence of MSG-induced AF.

To overcome the low inclusion rate, the study was initially prolonged from a, beforehand anticipated, one-year period to a four-year period, but it was eventually terminated for practical and financial reasons. Legislation prohibited us to buy MSG at the supermarket for €4.19 per kg. Instead, MSG and placebo tablets were produced by our hospital’s pharmacist at a cost of approximately €15,000 and with a strict perishable date. The delay in medical ethical approval and manufacturing of study medication raised questions among some of the motivated study participants about an alliance between pharmacists and MSG manufacturers. Legislation indeed lead to pre-term termination of the study and thus demonstrated to have been an effective strategy to boycott study finalisation.

Although only six participants were included, we could perform a robust analysis. We included only self-reported MSG-induced AF to increase the likelihood of AF occurrence and to determine if a causal relation exists. The administered boluses of 1.5 g and 3 g MSG can be considered to be high doses, as an average European consumes 0.3–0.5 g MSG per day. This is remarkably less than in Asian countries, where the daily intake is around 1.2–1.7 g MSG [10].

As a violation of the protocol, one participant stopped by a Chinese restaurant, likely to increase his already high dose of 3 mg MSG, but no AF occurred. Moreover, one of the two reported AF episodes after MSG intake occurred during night-time and was attributed to romantic activity. The imbalance between the attributions to the syndrome and the occurrence of MSG-induced AF could be explained by suggestive questioning. Kerr et al. found that suggestive questioning in a food-symptomatology survey raised the percentage of respondents believing to be susceptible to the Chinese restaurant syndrome from 6.6 to over 30 per cent [11]. The current study focussed on arrhythmias, but a wide range of symptoms has been associated with the Chinese restaurant syndrome. Recurrent symptoms such as sleeplessness or agitation after MSG intake could be mistaken for AF onset. This could further be endorsed by suggestive questioning or information from Internet websites.

Unfortunately, we were unable to assess MSG consumption as part of a Chinese meal in the appropriate setting. A very important trigger for AF onset after a Chinese dinner may be the concomitant non-alcoholic and alcoholic consumptions. It is well plausible that an MSG-enriched Chinese meal is accompanied by a large intake of water. This fluid challenge may cause AF. Moreover, a Chinese dinner may taste better when the spicy flavour is washed away with alcohol. Alcohol consumption has indeed been associated with AF [3]. Lastly, it can be hypothesised that the notorious big servings of the Chinese restaurant trigger AF through a vagal response caused by a full stomach.

## Conclusion

We cannot proof that MSG is associated with AF in self-reported MSG-sensitive patients. Future research is recommended to assess the effect of MSG on the diverse symptoms of the Chinese restaurant and should include qualitative assessments.

Readers may rest assured that the already prepared Christmas dinner with products containing MSG is safe for consumption. We do strongly recommend staying off the high dose of 3 g MSG though, as the flavour effect may be overwhelming (a recipe of *Peking duck* is provided in the online supplementary material under B).

## Caption Electronic Supplementary Material

Supplementary A. Study inlcusion and exclusion criteria; Supplementary B. Recipe of Peking duck
