# Supplementary material for: Can we spice up our Christmas dinner? Busting the myth of the ‘Chinese restaurant syndrome’
Source: Neth Heart J. 2017 Nov 10;25(12):664–8. doi: 10.1007/s12471-017-1053-5 (PMC5691820; doi:10.1007/s12471-017-1053-5)
Supplement: Supplementary file 1 — Supplementary A. Study inlcusion and exclusion criteria; Supplementary B. Recipe of Peking duck [file 12471_2017_1053_MOESM1_ESM.doc]

# Supplementary Material

# Supplementary material A.

# Table 1 Inclusion and exclusion criteria

| Inclusion Criteria | Exclusion Criteria |
| --- | --- |
| Written informed consent | Myocardial infarction (defined as CKMB> twice upper limit of normal) within the preceding 2 months |
| Reported MSG-triggered AF | NYHA class IV/IV heart failure symptoms, or class II-III with a recent decompensation requiring hospitalisation (unless related to or aggravated by AF) |
| Age: 18 and 80 years | Cerebrovascular accident (defined as any sudden neurological deficit lasting longer than 24 hours, with or without pathological changes on the CT cerebrum) with the preceding 6 months |
| Symptomatic, paroxysmal AF | Pregnancy or childbearing potential without adequate contraception |
| AF was documented on ECG, Holter or pacemaker electrocardiogram at least once in the 6 months preceding presentation | Requirement of antiarrhythmic medication for ventricular arrhythmias. |
| In sinus rhythm at the moment of study inclusion | Left ventricular ejection fraction <30% |

*CKMB*

# Supplementary material B.

# Recipe of Peking Duck

We thank the owners and cooks from the *New King* restaurant in Amsterdam, who were so kind to provide us with their original recipe of Peking duck.

**Peking Duck**

1. Buy a complete duck, we use 3300 grams ourselves. The duck may be frozen, but be sure it is completely thawed before you start.
2. Open up the duck at the tail end (10-15 cm).
3. Make sure to remove all organs.
4. Rinse the inside of the duck thoroughly. Make sure not to damage the outer skin.
5. Add 2 tablespoons of a mixture of the following spices: sugar, white pepper, five-spice powder, ginger powder, ***Ve-tsin***.
6. Add 3 tablespoons of hoisin sauce.
7. Add 2 whole star anise pods.
8. Add 3 bay leaves.
9. Now inflate the duck with a pump until the duck becomes nicely round.
10. Stich up the back of the duck.
11. Submerge the duck 3x in a large pan with boiling water.
12. Fill a bucket with 50% water and 50% vinegar. Add maltose to the water-vinegar mixture.
13. Submerge the duck in the water-vinegar mixture for the classic brown-stained color.
14. Dry the duck for 8 hours. You may use a fan to speed up the drying process.
15. Put the duck in a preheated oven for 1 hour at approximately 220 (depends on the type of oven used).
16. Enjoy your Christmas dinner!
